# Localization of human adipose-derived stem cells and their effect in repair of diabetic foot ulcers in rats

**DOI:** 10.1186/s13287-016-0412-2

**Published:** 2016-10-22

**Authors:** Rongfeng Shi, Yinpeng Jin, Chuanwu Cao, Shilong Han, Xiaowen Shao, Lingyu Meng, Jie Cheng, Meiling Zhang, Jiayi Zheng, Jun Xu, Maoquan Li

**Affiliations:** 1 Department of Interventional & Vascular Surgery, Shanghai Tenth People’s Hospital, Tongji University, School of Medicine, 301 Yanchang Road, Shanghai, 200072 People’s Republic of China; 2 Shanghai Liver Diseases Research Center, The Nanjing Military Command, Shanghai, 200235 People’s Republic of China; 3 Institute of Medical Intervention Engineering, Tongji University, 301 Yanchang Road, Shanghai, 200072 People’s Republic of China; 4 Department of Obstetrics & Gynecology, Shanghai Tenth People’s Hospital, Tongji University, School of Medicine, Shanghai, 200072 People’s Republic of China; 5 Department of Pathology, Shanghai Tenth People’s Hospital, Tongji University, School of Medicine, Shanghai, 200072 People’s Republic of China; 6 Advanced Institute of Translational Medicine, Tongji University, 1239 Siping Road, Shanghai, 200092 People’s Republic of China; 7 East Hospital, Tongji University, School of Medicine, Shanghai, 200092 People’s Republic of China

**Keywords:** Human adipose-derived stem cells, Diabetic foot ulcer, Tissue repair

## Abstract

**Background:**

Diabetic foot ulcer (DFU) is an intractable diabetic complication. Patients suffering from diabetes mellitus (DM) frequently present with infected DFUs. In this study, a wound healing model on diabetic rat foot was established to mimic the pathophysiology of clinical patients who suffer from DFUs. Our study aimed to explore the localization of human adipose-derived stem cells (hADSCs) and the role of these cells in the repair of foot ulcerated tissue in diabetic rats, and thus to estimate the possibilities of adipose-derived stem cells for diabetic wound therapy.

**Method:**

Sprague–Dawley rats were used to establish diabetic models by streptozotocin injection. A full-thickness foot dorsal skin wound was created by a 5 mm skin biopsy punch and a Westcott scissor. These rats were randomly divided into two groups: the hADSC-treated group and the phosphate-buffered saline (PBS) control group. The hADSC or PBS treatment was delivered through the left femoral vein of rats. We evaluated the localization of hADSCs with fluorescence immunohistochemistry and the ulcer area and ulcerative histology were detected dynamically.

**Result:**

The hADSCs had a positive effect on the full-thickness foot dorsal skin wound in diabetic rats with a significantly reduced ulcer area at day 15. More granulation tissue formation, angiogenesis, cellular proliferation, and higher levels of growth factors expression were also detected in wound beds.

**Conclusions:**

Our data suggest that hADSC transplantation has the potential to promote foot wound healing in diabetic rats, and transplantation of exogenous stem cells may be suitable for clinical application in the treatment of DFU.

**Electronic supplementary material:**

The online version of this article (doi:10.1186/s13287-016-0412-2) contains supplementary material, which is available to authorized users.

## Background

Diabetes mellitus (DM) is a universal health problem. According to the World Health Organization, approximately 171 million people suffered from DM globally in 2000 and this figure is projected to nearly double by 2030 [1]. It is estimated that between 15 and 25 % of patients suffering from DM develop foot ulcers during their lifetime, which seriously affect their quality of life, can be life-threatening, and are responsible for the majority of hospital admissions among diabetics [2]. Diabetic foot ulcers (DFUs), which pose substantial risks of seriously infection and amputation, constitute a major public health burden in both the developed and developing world [3, 4]. The 5-year mortality rate associated with DFUs requiring amputation ranges from 39 to 80 %, extremely close to the most aggressive forms of cancer [5, 6]. Therefore, novel methods that promote healing in diabetic foot disease are continuously being investigated to reduce the morbidity and mortality.

Recent studies have reported that mesenchymal stem cell (MSC) therapy may accelerate wound healing. Stem cells represent a type of undifferentiated cells, which could be expanded extensively in vitro [7]. Adult stem cells have prolonged self-renewal potency with the ability to proliferate and differentiate into various cell types. Bone marrow-derived mesenchymal stem cells (BMSCs) are an important source of adult stem cells which have been demonstrated to contribute to the reconstitution of the dermal fibroblast population and enhance wound healing [8]. Adipose-derived stem cells (ADSCs) are another important source of adult stem cells with characteristics similar to BMSCs, including cell surface markers, gene expression profile, immunosuppressive properties, and differentiation capacity [9–11]. Moreover, ADSCs have several advantages including ease of isolation, rapidity of expansion, and less donor morbidity compared with BMSCs [12, 13]. ADSCs have therefore been applied as an attractive cell source for cell transplantation therapy in regenerative medicine, which might be applied to the treatment of DFU [14, 15]. However, extensive preclinical studies are needed to evaluate the treatment potential of ADSCs for DFU, and the underlying mechanisms of cellular repair in DFUs should be elucidated.

In the present study, we transplanted human ADSCs (hADSCs) via the left femoral vein in the rat model of diabetes, and examined their effects on wound healing compared with vehicle control medium. The mobilization and localization of transplanted hADSCs to DFUs were detected using the lentivirus expressing ZsGreen. To further explore the potential mechanisms of repairing DFUs, we analyzed the inflammatory factors and the growth factors in the foot ulcerative tissue. We found that hADSCs localized superficially in ulcerative tissue, reducing inflammation, promoting the release of growth factors, and stimulating vasculogenesis and tissue repair in DFUs. The obtained data provided important information for the potential application of ADSC transplantation for DFU treatment.

## Methods

### Animals and model for diabetic foot ulceration

Adult female Sprague–Dawley rats (150–200 g, grade: clean, license: SCXK 2013-0005) at the age of 4–6 weeks were purchased from SLAC Laboratory Animal Co., Ltd (Shanghai, China) and used to establish a rat model for diabetes. All rats were maintained on a 12-hour light cycle in the animal facility of the Animal Unit of Tongji University. All animal experiments were conducted in accordance with the National Institutes of Health Guide for the Care and Use of Laboratory Animals and approved by the Biological Research Ethics Committee of the Chinese Academy of Sciences. Diabetes was induced in rats with streptozotocin injected intraperitoneally once at a dose of 100 mg/kg (in 0.01 M sodium citrate, pH 4.3). The blood glucose level was checked daily and controlled between16.7 and 33.3 mmol/l by 6–18 units/day of insulin (Wan-Bang Biochemical Medicine Co. Ltd, Xuzhou, China). After 4 weeks, a model for DFUs was created in rats as follows: anesthesia was induced with isoflurane inhalation, and a single round full-thickness skin wound was created on the dorsal hind foot of the diabetic rats by a disposable 5 mm skin biopsy punch and Westcott scissor.

### Isolation and culture of hADSCs

hADSCs were isolated from the discarded part of the transverse rectus abdominis musculocutaneous flap with patient consent in accordance with procedures approved by the Ethics Committee at the Chinese Academy of Medical Sciences and Peking Union Medical College. The fresh fat specimen was washed three times by phosphate-buffered saline (PBS) with 1 % penicillin/streptomycin and chopped by sterile operation scissors. The chopped tissues were digested with 0.2 % collagenase type I (Gibco, now part of Thermo Fisher Scientific, Waltham, MA, USA) at 37 °C for 1.5 hours. The suspension was neutralized with isometric culture media and centrifuged at 1500 rpm for 5 min. The cell pellet was then resuspended in Dulbecco’s modified Eagle’s medium/F12 (DMEM/F12) media (Gibco) supplemented with 10 % fetal bovine serum (FBS) (Gibco). Cells were plated at a density of 10^6^ cells/ml and then maintained at 37 °C in a humidified incubator supplemented with 5 % CO_2_, and the media was changed every other day. For continuous cell culture of hADSCs, the adherent hADSCs were washed with PBS and transferred into a Petri dish containing culture medium with 0.2 % trypsin–EDTA at 37 °C for 2 min. The cell suspension was transferred into a 15 ml tube and centrifuged at 1500 rpm for 5 min, the supernatant was removed, the pallet of hADSCs was resuspended in the 15 ml tube of culture medium, and 6.0 × 10^3^/cm^2^ of hADSCs were plated onto Petri dishes for continuous cell culture. The hADSCs at passage 4 were used for the following experiments.

### In-vitro adipogenic, chondrogenic, and osteogenic differentiation of hADSCs

The culture-expanded hADSCs were tested for their ability to differentiate into adipocytes, osteoblasts, and chondrocytes. Passage 4 hADSCs at a density of 5 × 10^3^ cells/cm^2^ were seeded into six-well plates precoated with a cover glass and induced for 3 weeks with the adipogenic (#HUXMA-90031; Cyagen Biosciences Inc., Guangzhou, China), osteogenic (#HUXMA-90021; Cyagen Biosciences Inc.), and chondrogenic (#HUXMA-90041; Cyagen Biosciences Inc.) differentiation media, respectively. The cells were then fixed in 10 % formalin and stained with Oil Red O, Alizarin red, or Alcian blue in accordance with the protocols of the manufacturer (Cyagen Biosciences Inc.). Stained cells were washed three times with PBS and detected using microscopy (IX71; Olympus, Tokyo, Japan).

### Detection of hADSC surface markers using immunofluorescent assay and flow cytometry

To observe the phenotypes, immunofluorescent assay and flow cytometry were employed simultaneously. Passage 4 hADSCs were seeded into a six-well plate and cultured for 24 hours, fixed for 30 min using 4 % paraformaldehyde, and incubated with CD29-FITC, CD44-PE, CD90-FITC, CD105-PE, CD34-FITC, and CD133-PE anti-human antibodies (0.5 μl/well) for 30 min. After three washes with PBS, the fluorescence of hADSCs was observed. For flow cytometry detection, passage 4 hADSCs were used to prepare a single-cell suspension, and subsequently 2 μl of CD29-FITC, CD44-PE, CD90-FITC, CD105-PE, CD34-FITC, and CD133-PE antibodies were added to the suspension. These cell aliquots were incubated for 30 min on ice photophobically, washed three times, and then detected using flow cytometry (BD FACSAria III; BD Biosciences, San Jose, CA, USA).

### Transfecting and tracing of transplanted hADSCs

Preparation of cell infection and lentiviral supernatants was performed as described previously [16]. Briefly, transient cotransfection of HEK293T cells were employed to produce the lentiviral vectors with four plasmids (Invitrogen). The vector-containing supernatants were harvested 48 and 72 hours after transfection, filtered, and then stored at −80 °C. hADSCs (passage 2) were infected with the lentivirus expressing ZsGreen at 70 % cell confluence [17]. Three days after transfection, the infection efficiency was detected using a fluorescence microscope (IX71; Olympus), and the cells were cultured successively. Twenty-four DFU model rats were injected with 5 × 10^6^ ZsGreen-hASDCs via the femoral vein, and at 24 hours, day 3 (D3), day 7 (D7), and day 15 (D15) the distribution and engraftment of hADSCs in the wound bed were detected dynamically using fluorescence immunohistochemistry analysis with the antibodies specific against ZsGreen (Abcam Ltd, Cambridge, UK).

### Transplantation of hADSCs to diabetic rats

Thirty diabetic model rats with foot ulceration were successfully duplicated and randomly divided into a hADSC-treated group and a PBS control group. We bluntly dissected the left femoral vein and injected 1 ml of hADSC suspension (5 × 10^6^ cells) via the femoral vein and the control group was injected with 1 ml of PBS. Local hemostasis and wound suturation were then performed. At D3, D7, and D15 after transplantation of hADSCs, the rats were sacrificed and the feet with diabetic ulcers were harvested. The feet were cut off vertically along the center line of the ulceration and divided into two equal parts. Half of each foot was stored immediately in liquid nitrogen to survey the inflammatory factors and the growth factors by proteome profiler array and enzyme-linked immunosorbent assay (ELISA). The other half was fixed in 4 % phosphate-buffered paraformaldehyde for histological assessment and immunohistochemical analysis.

### Measurement of ulceration contraction rate

Before harvest, the size of the wounded area measured with a ruler was recorded on parfocal digital photographs taken of each rat’s foot at D3, D7, and D15 after injecting the corresponding preparations (PBS for control group and hADSCs for experimental groups). A digital camera (DMC-LX5GK; Panasonic, Japan) was employed to take pictures and the ulcer area was analyzed by Image-Pro Plus 4.5 software [18].

### Histological assessment of DFU healing

Half of the ulcerative tissue was collected from rat feet in both groups, fixed in 4 % phosphate-buffered paraformaldehyde, embedded in paraffin, and sectioned at 4.0 μm. The sections were dehydrated with successive concentrations of ethanol and washed twice in distilled water. The sections of the ulcerative tissue at D15 were stained with hematoxylin and eosin (H&E) and with Masson’s trichrome in accordance with the protocols of the manufacturer (Cyagen Biosciences Inc.) to detect the re-epithelialization/granulation tissue formation and collagen deposition, respectively. Analogously, the status of cell apoptosis and proliferation in the diabetic ulcerative tissue were detected using the terminal deoxynucleotidyl transferase dUTP nick end labeling (TUNEL) assay and Ki67 immunofluorescence staining (DAKO, CA, USA), respectively. Finally, the histological sections were observed and analyzed under a microscope (Leica DMR 3000; Leica Microsystem) by two blinded experienced investigators.

### Vessel density assessment

For microvessel density determination, the ulcerative tissue was analyzed using immunostaining with mouse anti-CD31 primary antibody. Briefly, after fixing, embedding in paraffin, and dewaxing, the tissue sections were blocked in 3 % normal goat serum/0.3 % Triton X-100/0.1 % BSA (Sigma Aldrich) in PBS. The sections were then incubated for 24 hours at 4 °C with the primary antibody (primary) against CD31 (1:100 dilution, sc-53526; Santa Cruz), followed by goat anti-mouse IgG (secondary) for 1 hour at room temperature. After hematoxylin staining, tissue sections were washed and then dehydrated with ethanol, treated with dimethylbenzene, and sealed for microscopic analysis by two blinded experienced investigators. The number of CD31-positive vessels was quantified using Image-Pro Plus 4.5 software across five nonconsecutive tissue sections for each wound.

### Detection of growth factors and inflammatory factors

The growth factors and inflammatory factors were detected by ELISA and proteome profiler array. The other half of the tissue harvested from foot ulcers was cut into smaller pieces and washed in sterile PBS. Total protein was extracted, centrifuged, and resuspended in sample application buffer containing a protease inhibitor cocktail (Promega, Madison, WI, USA). The levels of growth factors and inflammatory factors in the foot ulcerative tissue were measured using the enzyme ELISA kit (Shanghai ExCell Biology, Inc., Shanghai, China) and proteome profiler array (R&D Systems, Inc., USA) in accordance with the respective product instruction manual. For growth factors, the optical density of each well was determined by spectrophotometer under a 450 nm wavelength after a substrate solution of hydrogen peroxide was added to the plate, and the concentrations of cytokines were calculated. For inflammatory factors, pixel densities on developed X-ray films were collected and analyzed using Image-Pro Plus 6.0 image analysis software (Media Cybernetics Inc, Silver Spring, MD, USA).

### Statistical analysis

Data are expressed as mean ± SD of at least three independent experiments. Differences between experimental groups were assessed by Student’s *t* test or one-way analysis of variance (ANOVA) followed by Dunnett’s test. For all statistical analyses, *P* < 0.05 was considered to be statistically significant.

## Results

### Characterization of hADSCs for transplantation

**Fig. 1:**
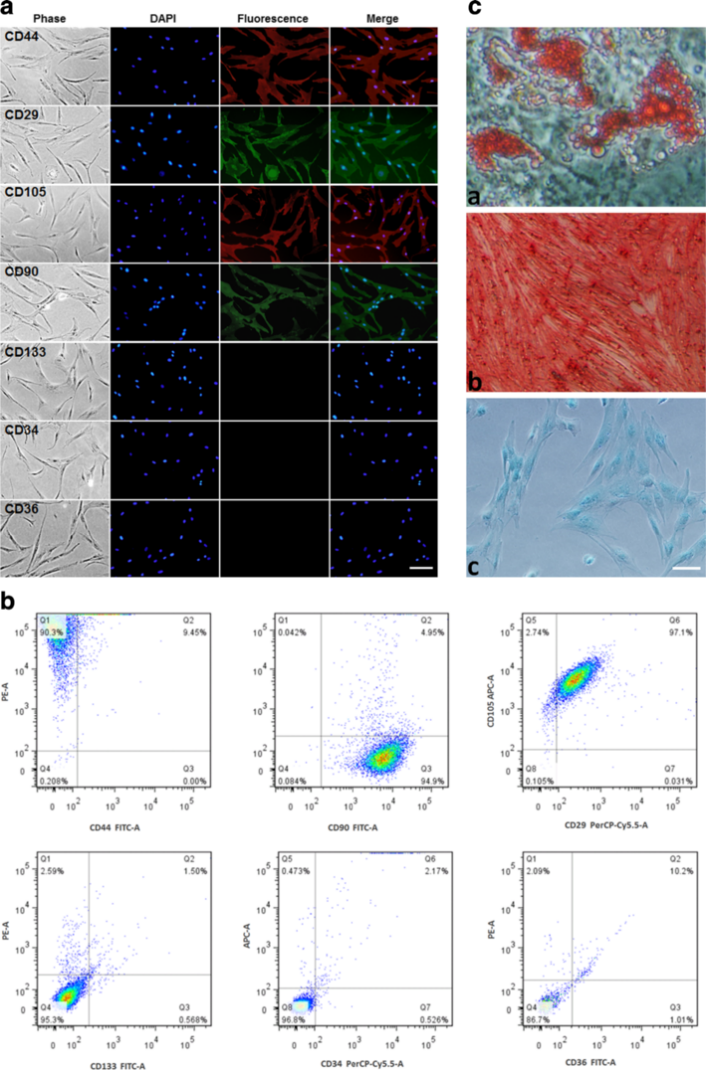
Characterization of hADSCs. **a** Immunofluorescent assay indicated that ADSCs were positive for cell surface markers CD44, CD29, CD105, and CD90 and were negative for CD133, CD34, and CD36. *Scale bar*, 50 μm. **b** Flow cytometry also revealed that CD29, CD44, CD90, and CD105 were highly expressed and CD133, CD34, and CD36 were negligible for the cells. **c** Triplet differentiation assays revealed that ADSCs could differentiate into adipocytes (*a*), osteocytes (*b*), and chondrocytes (*c*), which were stained by Oil Red O, Alizarin red, and Alcian blue, respectively. *Scale bar*, 25 μm

After four passages in culture, the expanded hADSC population became homogeneous, showing a monolayer of adherent cells, demonstrating a typical fibroblast-like and shuttle-shaped morphology. To characterize the phenotype of hADSCs, immunofluorescent assay and flow cytometry analysis were performed. The results revealed that CD44, CD29, CD105, and CD90 were signally expressed, whereas the cells expressed negligible levels of CD133, CD34, and CD36 for immunofluorescent assay (Fig. 1a). Furthermore, flow cytometry revealed more than 90 % of cells strongly expressed surface antigens such as CD44 (90.3 %), CD90 (94.9 %), CD29 and CD105 (97.1 %) but were virtually negative for CD133, CD34, and CD36 (all <3 %), which were consistent with the outcome of the immunofluorescent assay (Fig. 1b). To definitively characterize the multipotency of hADSCs, a triplet differentiation assay was performed, which included adipogenic differentiation, osteogenic differentiation, and chondrogenic differentiation. After culture in adipogenic induction media for 3 weeks, more than 80 % of cells differentiated into adipocytes that could be stained with Oil Red O. Similarly, after osteogenic and chondrogenic induction, more than 70 % of cells differentiated into osteoblasts and chondrocytes as demonstrated by Alizarin red staining and Alcian blue staining, respectively (Fig. 1c). The results of triplet differentiation assay indicated that the multipotency of cultured hADSCs was well maintained.

### Macro-evaluation of ulceration healing

**Fig. 2:**
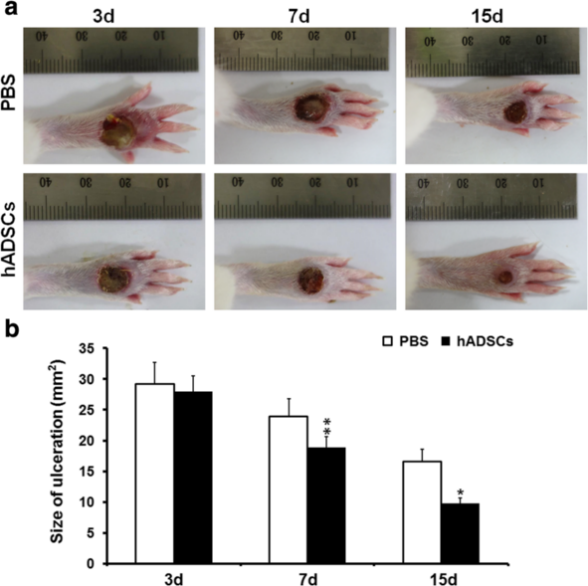
Diabetic foot ulceration healing at different time points after treatment in the hADSC-treated and control groups. **a** Representative images of ulcerations on diabetic rat feet at D3, D7, and D15 after treatment with hADSCs and PBS. **b** Mean area of diabetic foot ulceration in hADSC-treated model rats during observation compared with control model rats at D3, D7, and D15 after treatment. Averaged data presented as mean ± SD. *P < 0.05, **P < 0.01. *d* days, *hADSC* human adipose-derived stem cell, *PBS* phosphate-buffered saline. Representative images and mean area of the foot ulcers in the euglycemic rats group were shown in additional file 1

Thirty-six rats were successfully made into the model for DFU, and were randomly assigned to two groups. Half of the model rats (*n* = 18, treated group) were transplanted with hADSCs (5.0 × 10^6^ cells/rat) and the other half of the model rats (*n* = 18, control group) received the same volume of PBS via the femoral vein. At D3, D7, and D15 after treatment, the rats with DFUs were sacrificed (six rats in each group at every time point). All rats in both the treated group and the control group showed no adverse reactions, with no significant body weight changes during the 15-day experimental period. The blood glucose level was checked every day, and there was no significant difference between the two groups (26.9 ± 2.5 mmol/l in the control group vs 28.1 ± 3.1 mmol/l in the treated group, *P* > 0.05). The representative ulceration images for the two groups at D3, D7, and D15 post treatment are presented in Fig. 2a. Figure 2b shows the mean area of ulceration at relevant time points. At D3 post treatment, incrustation was formed in both groups, and the size of the foot ulcers were somewhat enlarged in the both groups (29.2 ± 3.2 mm^2^ in the control group vs 27.9 ± 2.9 mm^2^ in the treated group), which might be due to the intense inflammatory reaction. At D7, the size of the foot ulcers was significantly reduced in the group that received hADSCs (23.9 ± 2.8 mm^2^ in the control group v. 18.9 ± 1.9 mm^2^ in the treated group, *P* < 0.01). At D15, diabetic rat foot ulcers in the rats treated with hADSCs were further reduced and tended to be nearly healed (16.6 ± 2.1 mm^2^ in the control group vs 9.8 ± 1.1 mm^2^ in the treated group, *P* < 0.05). Our data demonstrate that transplanted hADSCs could improve foot ulcer healing in diabetic model rats.

### Histological assessment of wound healing

**Fig. 3:**
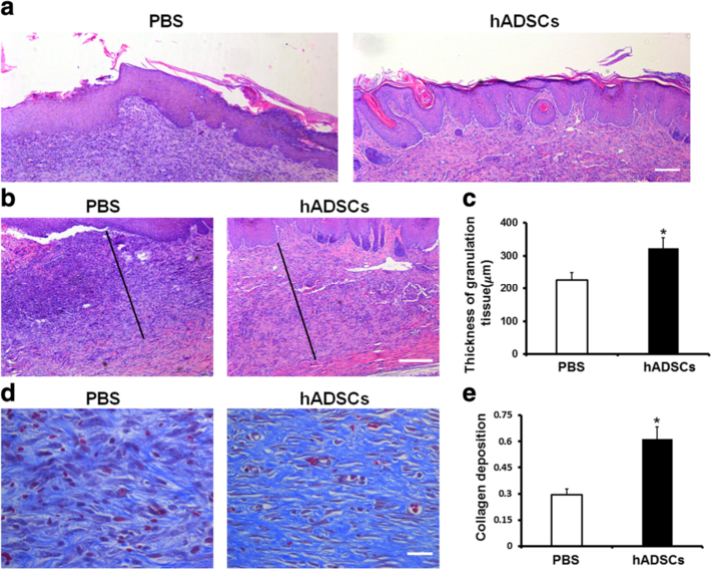
Effects of hADSCs on the epithelialization and granulation tissue regeneration at D15 after treatment. **a** H&E staining of sections showed better dermal re-epithelialization on the foot ulcers in model rats treated with hADSCs compared with control model rats. *Scale bar*, 100 μm. **b** Granulation tissues in the hADSC-treated group were thicker than those in the PBS-treated group. *Scale bar*, 100 μm. **c** Statistical thickness of granulation tissues by computer-assisted morphometric analysis. **d** Collagen deposition assessed by Masson Trichrome staining. The hADSC-treated group showed more intense blue staining than the PBS-treated group, suggesting that hADSC treatment accelerated collagen deposition in the granulation tissues. *Scale bar*, 50 μm. **e** Quantification analysis of the Masson Trichrome staining section by digital image analysis. Averaged data presented as mean ± SD. *P < 0.05. *hADSC* human adipose-derived stem cell, *PBS* phosphate-buffered saline

**Fig. 4:**
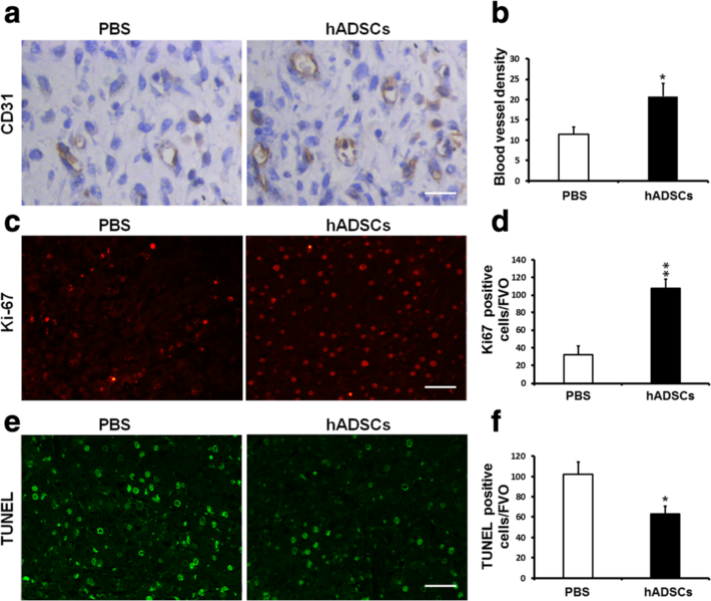
Effects of hADSCs on angiogenesis, cell proliferation, and apoptosis of wounds at D15 after treatment. **a** Immunohistochemical staining of CD31 showed more blood vessel structures on the wound bed in model rats treated with hADSCs were clearly increased compared with control model rats. *Scale bar*, 50 μm. **b** Quantification of blood vessel density expressed as the average number of CD31^+^ vessels per high-power field. **c** Cellular proliferation on the wound bed observed by Ki-67 immunofluorescence staining. The hADSC-treated group showed more positive cells than the PBS-treated group, indicating that hADSC treatment promoted cell proliferation in DFU healing. *Scale bar*, 100 μm. **d** Quantification analysis of the Ki-67 staining by digital image analysis. **e** Apoptotic cells detected by TUNEL assays. Few apoptotic cells were detected in the hADSC-treated group, suggesting that hADSC treatment reduced the apoptosis in the process of healing. *Scale bar*, 100 μm. **f** Quantification of TUNEL immunofluorescence staining by digital image analysis. Averaged data presented as mean ± SD. *P < 0.05, **P < 0.01. *hADSC* human adipose-derived stem cell, *PBS* phosphate-buffered saline, *TUNEL* terminal deoxynucleotidyl transferase dUTP nick end labeling

Histological analysis of the wound bed was performed to further evaluate the healing of the foot ulcers. The histological observation demonstrated that the tissue regeneration was much greater in the group treated with hADSCs compared with the PBS group. H&E-stained skin sections from the wound bed at D15 after treatment showed that wounds were not fully re-epithelialized in the control group; however, nearly complete re-epithelialization was achieved in the hADSC-treated group (Fig. 3a). Furthermore, the granulation tissue in the hADSC-treated group was significantly thicker than that in the control group (Fig. 3b, c: 332.8 ± 31.8 μm vs 226.6 ± 27.5 μm, *P* < 0.05). Collagen formation in the callus at D15 after treatment was assessed by Masson trichrome staining using computer-assisted morphometric analysis. Figure 4c, e show the presence of a large amount of collagen deposition organized in aligned fibers in the hADSC-treated rats, but relatively less in the PBS-treated rats. The mean density values of the hADSC-treated group was 0.615 ± 0.047, and for the control group was 0.295 ± 0.026 (*P* < 0.05). These findings indicate that hADSCs significantly accelerated epithelialization, formation of granulation tissue, and collagen deposition, and thus promoted wound healing of the DFUs in rats.

### Effects of hADSC treatment on angiogenesis, cell proliferation, and apoptosis

Because angiogenesis is a critical factor for skin wound healing, the vascularity of wound granulation tissue was evaluated. The small blood vessels of the wounds were characterized by CD31 immunohistochemical staining of 4 μm tissue sections at D15 to detect neovascularization. We found that large and dense vessels were growing widely in wound granulation tissue of the hADSC-treated group. However, only puny and narrow new vessels at the wound bed were presented in the control group. There was a significant increase in the density of CD31-positive capillary lumens in the hADSC-treated wounds and the hADSC transplantation significantly enhanced the wound angiogenesis (Fig. 4a, b). Given that the cellular proliferation in the wound tissues may contribute to the ulcer healing, we next investigated whether the hADSC transplantation promoted cellular proliferation by Ki-67 staining. The Ki67-positive cells were distributed diffusely in the basal layer of the epidermis of the hADSC-treated group but less in the control group. The mean density of Ki67 expression in the hADSC-treated groups at D15 was significantly higher compared with the control group (Fig. 4c, d). TUNEL assays were used to detect whether hADSC treatment reduced the apoptosis. The tissue sections of the control group were distinctly characterized by apoptotic nuclei cells, indicative of cellular apoptosis, whereas fewer apoptotic cells were observed in the hADSC-treated group, and quantification analysis revealed that approximately 40 % of apoptotic cells were reduced in the hADSC-treated group compared with the control group (Fig. 4e, f). These results demonstrated that hADSC transplantation promoted cellular proliferation and reduced apoptosis simultaneously in the process of ulcer healing.

### Fate of hADSCs after transplantation

**Fig. 5:**
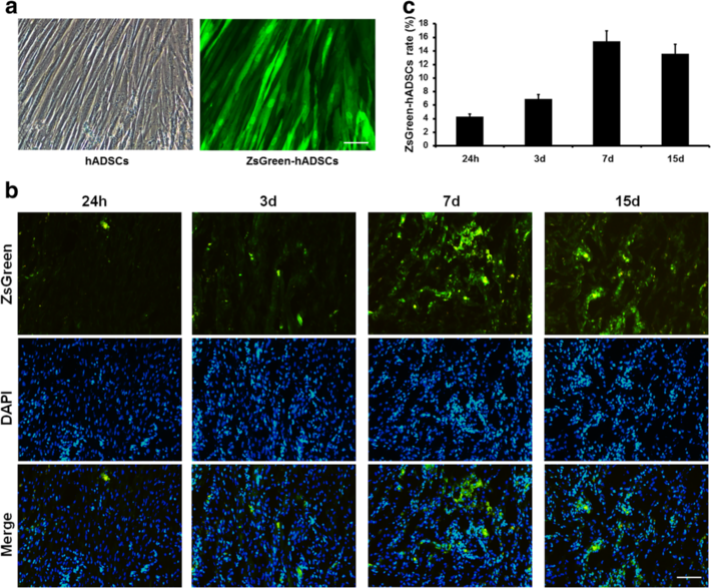
Fate of hADSCs after transplantation in DFUs. **a** hADSCs were labeled by the lentivirus expressing ZsGreen and detected under bright-field and green fluorescence. *Scale bar*, 25 μm. **b** Images of transplanted ZsGreen-positive cells at different time points in DFUs. The rate of ZsGreen-positive cells was gradually increased during the first week after transplantation, with the highest intensity on D7. *Scale bar*, 100 μm. **c** Rate of ZsGreen-positive cells at different time points. *d* days, *h* hours, *hADSC* human adipose-derived stem cell

To trace the distribution of transplanted hADSCs in foot ulcer tissues of the diabetic rats, we employed the lentivirus expressing ZsGreen to infect the hADSCs and injected it via the femoral vein. The labeling efficiency of the hADSCs was more than 80 %, and their corresponding progenies were traced using a fluorescence microscope at 488 nm (Fig. 5a). The frozen sections of ulcer tissues were made at 24 hours, D3, D7, and D15 after transplantation, and the intensity and distribution of green fluorescence were detected immediately. The number of ZsGreen-positive cells in these fields was counted by two observers blinded to the observational group and total cell numbers in these fields were also counted. The ZsGreen-positive cells were observed in the ulcer tissues 24 hours after hADSC transplantation, suggesting that transplanted cells could rapidly reach the injury skin tissues. Moreover, we also found ZsGreen-positive cells in the ulcer tissues at D3, D7, and D15 after transplantation (Fig. 5b). The rate of ZsGreen-positive cells was gradually increased during the first week after transplantation, and the highest intensity was observed at D7 with a rate of 15.1 %. As time went on, the intensity was slightly decreased at D15. Nonetheless, a considerable quantity of ZsGreen-positive cells (13.6 %) survived in the ulcer tissues (Fig. 5c). Our data demonstrated that the hADSCs transplanted via the femoral vein had the ability to migrate to the wound area to participate in the whole wound healing process.

### Variation of growth factors and inflammatory factors after hADSC treatment

**Fig. 6:**
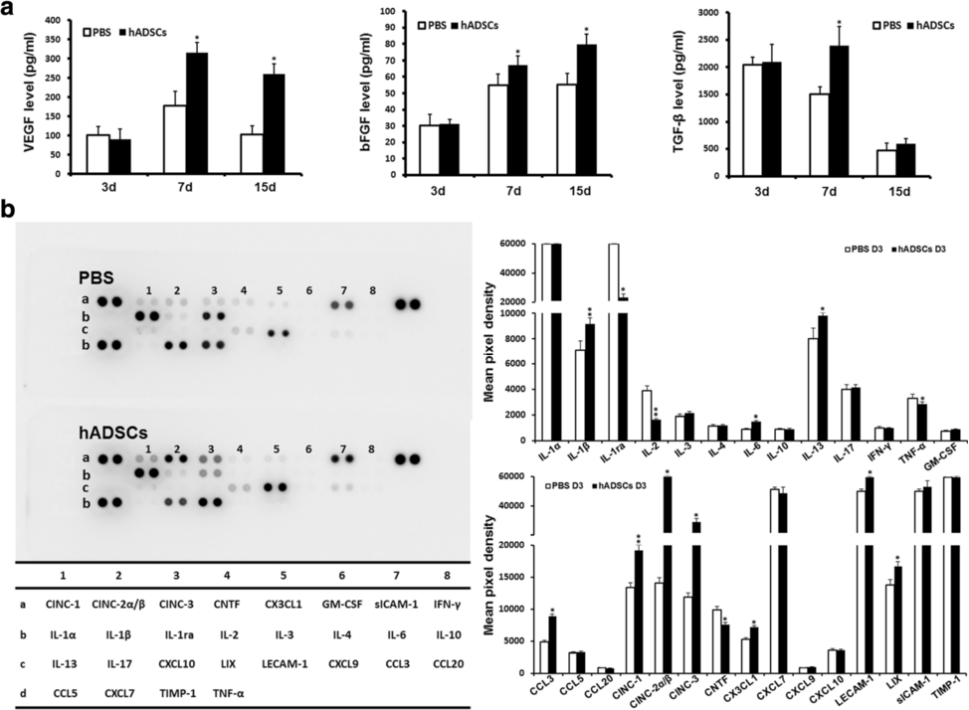
Effect of hADSC treatment on key growth factors and inflammatory factors in the DFU tissues. **a** Levels of VEGF, bFGF, and TGF-β in the DFU tissues at different time points were determined by enzyme-linked immunosorbent assay (ELISA).The level of VEGF, bFGF, and TGF-β was obviously higher in the hADSC-treated group compared with the PBS-treated group on D7 but there were no significant differences on D3.The higher level of VEGF and bFGF continued to D15 after treatment. **b** The R&D Systems Rat Cytokine Antibody Proteome Profiler Array system was used to screen for the inflammation-related cytokines and chemokines in the DFU tissues on D3 after treatment. Quantification of cytokines and chemokines mean pixel density was analyzed by Image-Pro Plus 6.0. *P < 0.05, **P < 0.01. *d* days, *hADSC* human adipose-derived stem cell, *PBS* phosphate-buffered saline

To explore the potential correlation between particular growth factor levels and the wound healing process after hADSC treatment, the levels of VEGF, bFGF, and TGF-β were measured by ELISA. The growth factor levels in wound beds were obviously higher in the hADSC-treated group compared with the control group at D7 and D15 after treatment, especially VEGF and bFGF. However, at D3 these growth factors level did not show any difference between the two groups (Fig. 6a). The data were consistent with the process of area decrease in foot ulcers and the quantity of hADSCs migrated to the wound beds. Various cytokines and chemokines play critical roles in inflammation, and multiple factors compose a network in which one cytokine is regulated by one or more other cytokines. A proteome profiler array was employed to detect the cytokines and chemokines in the ulcer tissues at D3, which allowed simultaneously analysis of 28 factors in a single experiment to investigate the multi-target strategy. After transplantation with hADSCs, multiple cytokines such as, IL-1ra, IL-2, TNF-α, and CNTF were significantly restrained, and the expressions of IL-1β, IL-6, IL-13, CCL3, CINC-1, CINC-2α/β, CINC-3, CX3CL1, LECAM-1, and LIX were significantly upregulated (*P* < 0.05 or *P* < 0.01), indicating that transplanted hADSCs could modulate the synthesis of several cytokines which were involved in the inflammatory process to inhibit inflammatory reaction (Fig. 6b).

## Discussion

Successful wound healing is a dynamic and complex process including a series of coordinated events: inflammation, cell migration, proliferation, differentiation, angiogenesis, fibroplasia, re-epithelialization, and tissue remodeling [19]. Many factors can impair wound healing in chronic wounds such as DFU, including infection, necrosis, tissue hypoxia, and excess levels of inflammatory cytokines, which prolong one or more phases of inflammation, proliferation, and remodeling [20]. Chronic lower extremity wounds have become some of the most common and severe complications of diabetes, and as the number of people diagnosed with diabetes continues to increase, so will the number of DFUs. During the past decades, many doctors and scientists have employed different approaches to promote ulcer healing, such as hyperbaric oxygen therapy [21], electrical stimulation [22], negative pressure wound therapy [23], and bioengineered skin [24]. However, there is not yet an ideal therapy that is widely acceptable. The ideal modality of wound healing in DFUs should have the synergistic effects of reducing inflammation, forming vital granulation tissue, and accelerating cellular proliferation and angiogenesis.

Stem cells are characterized by their ability to sustain self-renewal and multilineage differentiation and to form terminally differentiated cells under particular conditions [25]. These characteristics make stem cells ideal materials for renovation in tissue damage. Abundance in source, minimal invasion to acquire, efficacy, and safety for use are the ideal criteria of stem cell types for clinical use [26]. Wu et al. [27] have reported that the healing of wounds could be promoted through delivery of BMSCs, providing strong evidence that MSCs have the extraordinary plasticity and the ability to secrete growth factors which could accelerate wound healing. BMSCs are considered a benchmark for clinic purposes; however, BMSC isolation involves an invasive and painful procedure with the low yield [28, 29]. On the contrary, similar to BMSCs, ADSCs are also pluripotent cells that possess stem cell characteristics, such as extensive proliferation capacity, and differentiation potential to chondrocytes, adipocytes, neuron-like cells, and liver-like cells [30–32]. They present low immunogenicity and appear to be more genetically stable in long-term culture compared with BMSCs [33, 34]. Furthermore, ADSCs have several outstanding features and advantages described previously [12, 13]. Recent studies have shown that ADSC transplantation displays good therapeutic efficacy for various diseases, including diabetic skin ulcer [35]. Different to the ordinary diabetic ulcer, the pathogenesis of ulcer formation on the diabetic foot is more complex, usually accompanied with the pathological changes of peripheral vessel and nerve, and severe infection. In this respect, our data have demonstrated that hADSC transplantation could also efficiently accelerate the ulcer healing on the foot of diabetic rats. Because of the low immunogenicity, although we used hADSCs to transplant into immunocompetent rats instead of immunodeficient recipients, no obvious immunologic rejection reaction was found throughout the process. Many other researchers have also reported that they successfully transplanted human MSCs and human neural stem cells into immunocompetent rodent hosts and acquired functional outcomes [36, 37].

The potential of ADSCs that may benefit chronic wound healing include their ability to migrate to the site of inflammation and injury, participate in regeneration of damaged tissues, stimulate proliferation and differentiation of resident progenitor cells, promote angiogenesis and recovery of injured cells through growth factor secretion, facilitate matrix remodeling in the re-epithelialization phase, and exert immunomodulatory and anti-inflammation effects [38]. In this study, we successfully duplicated the diabetic rat of foot ulceration model and transplantation with the hADSCs. The results demonstrated that these cells specifically targeted the foot ulcerative tissue within a short period of time after treatment with hADSCs, which was infected with the lentivirus expressing ZsGreen (Fig. 5). The localization of the cells associated with the hADSC transplantation was significant, and progressive restoration of normal tissue structure in the DFUs, with a dramatic reduction in the measured size of ulceration in hADSC-treated rats compared with the control rats, demonstrated a positive effect towards enhancing wound healing in diabetic rats. The main findings of this study are as follows: transplanted hADSCs particularly migrated to or found home in the wound tissues and contributed to tissue cells; transplanted hADSCs promoted re-epithelialization and granulation tissue formation; transplanted hADSCs significantly enhanced angiogenesis and cellular proliferation, and decreased cellular apoptosis; and transplanted hADSCs increased growth factor (VEGF, bFGF, and TGF-β) secretion and restrained the inflammation reaction. There is compelling evidence that ADSCs would be a powerful tool in regenerative medicine, suggesting the potential utility for the clinical management. The data collected from our study are consistent with the therapeutic effects of hADSCs reported previously on osteogenic defect [39] and Crohn’s disease [40]. In the current study, we not only validated the specific localization of hADSCs in diabetic foot ulcerative tissue in a rat model of diabetes, but also further explored more details of and the potential mechanisms involved in repair of DFU.

The key point in exploring the role of human stem cells for the treatment of DFUs in the rat model is to detect whether hADSCs participate in the restoration process. In the previous report, superparamagnetic iron oxide (SPIO) was employed to mark stem cells in combination with an MRI technique [41]. However, this method may not be suitable for long-term observation because of the feeble MRI signal after the progressive differentiation of stem cells in the target tissue. In our study, the ZsGreen carried by the lentivirus was used to mark the hADSCs and its labeling efficiency was approximately 90 %. ZsGreen is one of the members of the reef coral fluorescent protein (RCFP) family. The novel fluorescent proteins were isolated from nonbioluminescent species of reef-coral organisms. RCFPs do not require external cofactors or substrates and can be used in vivo or in vitro, with the same advantageous qualities that make GFP a suitable reporter. RCFP variants with brighter fluorescence and specific emission characteristics have been generated and are now commercially available [42]. Using this method, the fact that hADSCs specifically localized to the ulcer area in a time-dependent manner and significantly reduced the size of foot ulcers in diabetic rats treated with hADSCs compared with control rats was detected. Intravenously injected hADSCs can migrate through veins to sites of injury in response to potentially chemotactic signals to modulate inflammation and contribute to tissue remodeling. Previous studies found that implanted ADSCs were able to differentiate into keratinocytes, fibroblasts, and epithelial cells in the skin [38, 43, 44]. The ZsGreen-positive cells were observed in the ulcer tissues 24 hours after the hADSC transplantation, and the highest intensity was observed at D7. The underlying mechanisms may be related with progression of the wound healing process. The situation of ZsGreen-hADSCs injected via the femoral vein into the diabetic rat foot ulcers indicated that hADSCs were able to migrate to the wound area and participate in the ulcer healing process.

Both paracrine signaling and differentiation have been implicated as the mechanisms for ADSCs to improve tissue repair [45]. Factors secreted by ADSCs have been demonstrated to exhibit a variety of biological activities including facilitating skin wound closure [46] and suppressing immunoreaction [47]. As one of the most important findings of this study, we detected higher levels of VEGF, bFGF, and TGF-β in the foot ulcer tissues of rats in the hADSC-treated group compared with the control group. It has been widely recognized that VEGF is the most effective and specific growth factor that regulates angiogenesis, while bFGF is also an important growth factor in tissue regeneration because it affects migration and proliferation of fibroblasts, angiogenesis, as well as matrix deposition [48, 49]. Besides, TGF-β also possibly plays a pivotal role in healing process by stimulating cellular proliferation, differentiation, and ECM secretion and inhibiting inflammatory reaction [50]. The paramount importance of blood supply in the healing of wounds has long been appreciated. In the present study, increased capillary density of the wound bed in the hADSC-treated group was detected by immunohistochemical analysis. Meanwhile, thicker granulation tissue and complete re-epithelialization was achieved and the formation of new blood vessels is necessary to sustain and enhance newly formed granulation tissue. Granulation tissue is essential to wound healing, because it is formed on the surface of wounds to protect and provide nutrition to wounds, consisting of fibroblasts, new capillaries, and infiltrated inflammatory cells. Besides, a larger amount of collagen deposition, promoted cellular proliferation, and reduced apoptosis were found in the hADSC-treated group. These data were consistent with the higher level of growth factors we detected in the ulcer tissues. The application of these growth factors could increase capillary formation, collagen deposition, and the degree of granulation tissue. The results revealed that growth factors secreted by ADSCs played a particular and significant role in ADSC-mediated accelerated wound healing, while topical application of recombinant growth factors or cytokines directly to stimulate diabetic wound healing has been demonstrated to be without significant improvement because of the quick diffusion and drying in the open wound. Infections are frequent and disastrous complications in DM, and infections of various types may be more common and are more often severe in patients with DFUs [51]. Our data suggest that transplanted hADSCs could modulate the synthesis of several cytokines involved in the inflammatory process to inhibit the inflammatory reaction.

ADSCs are a particularly promising therapy for the treatment of chronic nonhealing wounds. In the field of wound healing, the possibilities of transplanted ADSCs might have greater significance in diabetic wounds, where there exists a complicated pathophysiology that we still do not understand completely. Our study showed that transplanted hADSCs particularly migrated to or found home in the wound tissues and created a biological microenvironment to accelerate healing by a variety of mechanisms. This comprehensive effect taken by ADSCs in chronic nonhealing wounds is a remarkable advantage that any previous single treatment could not achieve [21–24]. The more accurate mechanisms should be revealed clearly to develop more efficient treatment strategies. Long-term systemic effects of stem cell therapy have yet to be established, and the security of stem cell therapy should also be of concern. The hypothesis that the immunomodulatory effects of ADSCs may provide favorable conditions for tumor growth could be hard to detect with the use of stem cell in a short period of time [52]. Although no cases have been reported from patients treated with ADSC injections, long-term follow-up studies are necessary to rule out any negative side effects.

## Conclusions

In this study, transplanted hADSCs accelerated wound healing in the DFU model of rats, in part by modulating inflammation reaction and providing a source of growth factors that promote angiogenesis, cellular proliferation, and wound healing. Clinically, transplantation of ADSCs may represent a new strategy for improvement of foot ulcers in patients with diabetes.

## Additional file

Additional file 1: is a figure showing foot ulceration healing at different time points in euglycemic rats injected with PBS. (**A**) Representative images of ulcerations on the euglycemic rat feet at D3, D7, and D15 after injection. (**B**) Mean area of foot ulcerations on the euglycemic rat feet during observation at D3, D7, and D15 after injection (D3, 21.6 ± 2.3 mm^2^; D7, 16.9 ± 1.8 mm^2^; D15, 3.8 ± 0.5 mm^2^; *n* = 5 for each time point). (DOCX 187 kb)

## Abbreviations

BMSC: Bone marrow-derived mesenchymal stem cell
DFU: Diabetic foot ulcer
DM: Diabetes mellitus
ADSC: Adipose-derived stem cell
hADSC: Human adipose-derived stem cell
MSC: Mesenchymal stem cell
PBS: Phosphate-buffered saline
