# Supplementary material for: Localization of human adipose-derived stem cells and their effect in repair of diabetic foot ulcers in rats
Source: Stem Cell Res Ther. 2016 Oct 22;7:155. doi: 10.1186/s13287-016-0412-2 (PMC5075186; doi:10.1186/s13287-016-0412-2)
Supplement: Additional file 1: — is a figure showing foot ulceration healing at different time points in euglycemic rats injected with PBS. (A) Representative images of ulcerations on the euglycemic rat feet at D3, D7, and D15 after injection. (B) Mean area of foot ulcerations on the euglycemic rat feet during observation at D3, D7, and D15 after injection (D3, 21.6 ± 2.3 mm2; D7, 16.9 ± 1.8 mm2; D15, 3.8 ± 0.5 mm2; n = 5 for each time point). (DOCX 187 kb) [file 13287_2016_412_MOESM1_ESM.docx]

**Supplemental Materials**

**
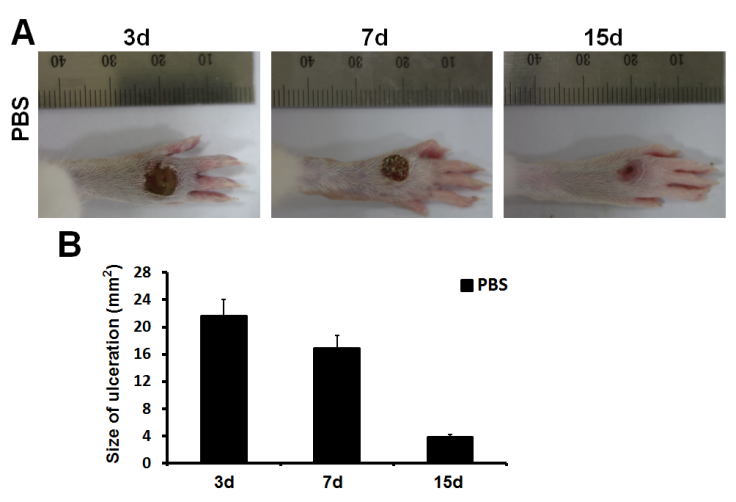
**

**Supplemental Figure Legend**

Foot ulceration healing at different time points on euglycemic rats injected with PBS. (A) Representative images of ulcerations on the euglycemic rat feet at 3, 7 and 15 days after injection. (B) The mean area of foot ulcerations on the euglycemic rat feet during observation at 3, 7 and 15 days after injection (D3 21.6 ± 2.3 mm^2^, D7 16.9 ± 1.8 mm^2^, D15 3.8 ± 0.5 mm^2^; n = 5 each time point).
